# Supplementary material for: Insight into the Anticancer Activity of Copper(II) 5-Methylenetrimethylammonium-Thiosemicarbazonates and Their Interaction with Organic Cation Transporters
Source: Biomolecules. 2020 Aug 20;10(9):1213. doi: 10.3390/biom10091213 (PMC7565988; doi:10.3390/biom10091213)
Supplement: Supplementary file 1 [file biomolecules-10-01213-s001.pdf]

Supplementary materials

Insight into the Anticancer Activity of Copper(II) 5-Methylenetrimethylammonium-Thiosemicarbazonates and Their Interaction with Organic Cation Transporters

Miljan N. M. Milunović [1,*], Oleg Palamarciuc [2], Angela Sirbu [2], Sergiu Shova [3], Dan Dumitrescu [4], Dana Dvoranová [5], Peter Rapta [5], Tatsiana V. Petrasheuskaya[6,7], Eva A. Enyedy [6,7], Gabriella Spengler [7,8], Marija Ilic [9,10,11], Harald H. Sitte [10], Gert Lubec [11] and Vladimir B. Arion [1,*]

[1] Institute of Inorganic Chemistry, Faculty of Chemistry, University of Vienna, Währinger Strasse 42, A-1090 Vienna, Austria

[2] Department of Chemistry, Moldova State University, A. Mateevici Street 60, MD-2009 Chisinau, Republic of Moldova; palamarciuco@gmail.com (O.P.); sirbuangela@yandex.ru (A.S.)

[3] Petru Poni Institute of Macromolecular Chemistry, Aleea Grigore Ghica Voda, Nr. 41A, 700487 Iasi, Romania; shova@icmpp.ro

[4] Elettra - Sincrotrone Trieste S.C.p.A, Strada Statale 14 - km 163,5 in AREA Science Park, 34149 Basovizza, Trieste, Italy; dan.dumitrescu@gmail.com

[5] Institute of Physical Chemistry and Chemical Physics, Faculty of Chemical and Food Technology, Slovak University of Technology in Bratislava, Radlinského 9, SK-81237 Bratislava, Slovakia; dana.dvoranova@stuba.sk (D.D.); peter.rapta@stuba.sk (P.R.)

[6] Department of Inorganic and Analytical Chemistry, Interdisciplinary Excellence Centre, University of Szeged, Dóm tér 7, H-6720 Szeged, Hungary; petrashev_tanya@tut.by (T.V.P.); enyedy@chem.u-szeged.hu (E.A.E)

[7] MTA-SZTE Lendület Functional Metal Complexes Research Group, University of Szeged, Dóm tér 7, H-6720 Szeged, Hungary; spengler.gabriella@med.u-szeged.hu

[8] Department of Medical Microbiology and Immunobiology, University of Szeged, Dóm tér 10, H-6720 Szeged, Hungary

[9] Department of Pharmaceutical Chemistry, Faculty of Life Sciences, University of Vienna, A-1090 Vienna, Austria; marija.ii.ilic@gmail.com

[10] Institute of Pharmacology, Centre for Physiology and Pharmacology, Medical University of Vienna, A-1090 Vienna, Austria; harald.sitte@meduniwien.ac.at

[11] Neuroproteomics, Paracelsus Private Medical University, post code Salzburg, Austria; gert.lubec@lubeclab.com

* Correspondence: miljan.milunovic@univie.ac.at (M.N.M.M.); vladimir.arion@univie.ac.at (V.B.A.)

Table of Contents	Page
Scheme S1. Reaction pathway to [H_2L^{1-4}]Cl and coper(II) complexes 1–4	3
Table S1-S3. Bond lengths and angles for 2	3–4
Figure S1–S17. ^{1}H, ^{13}C, HSQC and HMBC NMR spectra of [H_2L^R]Cl in [D_6]DMSO	5–13
Figure S18. UV/Vis absorption spectra of copper(II) complexes 1–4 in aqueous solutions	13
Figure S19. UV/Vis absorption spectra of [H_2L^{Me}]Cl and 2 in aqueous solution before and after partitioning, and in the n-octanol phase after the partitioning	14
Figure S20. Chemical shifts of the CH(4) proton of the major (●) and minor (●) isomers of [H_2L^H]Cl	14
**Figure S21.** Time-dependent UV/Vis absorption spectra of complex **1** in the presence of GSH before and after mixing their solutions in a tandem cuvette, and after the bubbling of oxygen	14
**Figure S22**. UV/Vis spectra measured during cyclic voltammetry of **2** in MeCN/*n*Bu_4NPF_6	15
**Figure S23**. Cyclic voltammograms of **1** and **3** in phosphate buffer solution + 0.1 M KCl (pH 7)	16
**Figure S24**. Cyclic voltammograms of **2** in DMSO/*n*Bu_4NPF_6	17
**Figure S25**. UV/Vis spectra of **2** in DMSO/*n*Bu_4NPF_6 measured during cyclic voltammetry	18

**Figure S26**. UV/Vis spectra of **3** in MeCN/*n*Bu$_4$NPF$_6$ measured upon cathodic reduction and anodic oxidation	19
**Figure S27.** Electronic absorption spectra of ABTS$^{•+}$ after mixing with **[H$_2$L^H]Cl**	20

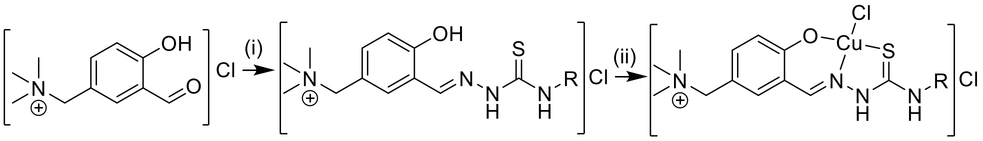

Scheme S1. Reaction pathway to **[H$_2$L$^{1–4}$]Cl** and copper(II) complexes **1–4**, R = H, Me, Et or Ph. Reaction conditions: (i) corresponding thiosemicarbazide, MeOH/H$_2$O = 1:3, 65 °C, 20 min. (ii) CuCl$_2$·2H$_2$O, H$_2$O, 50 °C.

Table S1. Bond lengths [Å] and angles [°] for **2.**

	**A**	**B**
Cu1-Cl1	2.2397(10)	2.2361(11)
Cu1-S1	2.2577(10)	2.2453(10)
Cu1-O1	1.910(2)	1.898(2)
Cu1-N1	1.960(2)	1.968(3)
S1-C8	1.706(3)	1.707(4)
O1-C1	1.305(4)	1.310(4)
N1-N2	1.380(4)	1.378(4)
N1-C7	1.294(4)	1.291(4)
N2-C8	1.338(4)	1.349(4)
N3-C8	1.317(4)	1.304(5)
N3-C9	1.453(4)	1.371(10)
N4-C10	1.533(4)	1.523(4)
N4-C11	1.494(5)	1.483(5)
N4-C12	1.490(5)	1.493(4)
N4-C13	1.497(5)	1.503(5)
C1-C2	1.401(4)	1.403(5)
C1-C6	1.427(4)	1.428(4)
C2-C3	1.383(5)	1.368(5)
C3-C4	1.401(5)	1.406(5)
C4-C5	1.370(5)	1.363(5)
C4-C10	1.504(4)	1.500(4)
C5-C6	1.413(4)	1.410(4)
C6-C7	1.420(4)	1.418(4)
N3-C9X	-	1.282(9)

	**A**	**B**
Cl1-Cu1-S1	91.41(4)	92.04(5)
O1-Cu1-Cl1	90.44(7)	91.26(8)
O1-Cu1-S1	175.84(7)	171.93(8)
O1-Cu1-N1	92.44(10)	92.18(10)
N1-Cu1-Cl1	176.49(8)	168.21(9)
N1-Cu1-S1	85.85(8)	86.06(8)
C8-S1-Cu1	97.25(11)	97.11(12)
C1-O1-Cu1	128.8(2)	129.1(2)
N2-N1-Cu1	117.53(19)	116.8(2)
C7-N1-Cu1	126.3(2)	126.4(2)
C7-N1-N2	116.1(3)	116.7(3)
C8-N2-N1	119.3(3)	119.3(3)
C8-N3-C9	123.9(3)	130.2(5)
C11-N4-C10	111.0(3)	110.7(3)
C11-N4-C13	109.1(3)	109.8(4)
C12-N4-C10	107.2(3)	107.8(3)
C12-N4-C11	109.6(3)	109.2(3)
C12-N4-C13	109.0(3)	108.6(3)
C13-N4-C10	110.8(3)	110.8(3)
O1-C1-C2	119.4(3)	119.1(3)
O1-C1-C6	123.3(3)	123.4(3)
C2-C1-C6	117.3(3)	117.5(3)
C3-C2-C1	121.7(3)	121.7(3)
C2-C3-C4	121.3(3)	121.3(3)
C3-C4-C10	119.8(3)	120.9(3)
C5-C4-C3	118.1(3)	117.9(3)
C5-C4-C10	122.1(3)	121.1(3)
C4-C5-C6	122.2(3)	122.8(3)
C5-C6-C1	119.4(3)	118.7(3)
C5-C6-C7	116.6(3)	117.6(3)
C7-C6-C1	124.0(3)	123.6(3)
N1-C7-C6	125.1(3)	125.2(3)
N2-C8-S1	120.0(2)	119.6(3)

N3-C8-S1	122.7(2)	122.0(3)
N3-C8-N2	117.3(3)	118.4(4)
C4-C10-N4	115.3(3)	115.3(3)

Table S2. Bond lengths [Å] and angles [°] for **3**.

**Cu1-Cl1**	**2.240(2)**	**N4-C11**	**1.466(9)**
Cu1-S1	2.245(2)	N4-C12	1.491(8)
Cu1-O1	1.899(5)	N4-C13	1.500(8)
Cu1-N1	1.979(5)	C1-C2	1.408(10)
S1-C8	1.697(8)	C1-C6	1.405(9)
O1-C1	1.311(8)	C2-C3	1.369(9)
N1-N2	1.370(7)	C3-C4	1.390(9)
N1-C7	1.285(8)	C4-C5	1.365(9)
N2-C8	1.354(9)	C4-C10	1.497(8)
N3-C8	1.317(9)	C5-C6	1.434(8)
N3-C9	1.465(9)	C6-C7	1.433(8)
N4-C10	1.533(7)	C9-C14	1.477(12)
			[1] $1-x, +y, {}^{3}/_{2}-z$

Cl1-Cu1-S1	89.72(8)	O1-C1-C2	119.1(6)
O1-Cu1-Cl1	91.74(16)	O1-C1-C6	124.1(6)
O1-Cu1-S1	175.94(15)	C6-C1-C2	116.8(6)
O1-Cu1-N1	92.5(2)	C3-C2-C1	122.5(6)
N1-Cu1-Cl1	171.61(15)	C2-C3-C4	121.3(6)
N1-Cu1-S1	86.51(17)	C3-C4-C10	120.5(6)
C8-S1-Cu1	97.0(3)	C5-C4-C3	117.9(6)
C1-O1-Cu1	128.4(4)	C5-C4-C10	121.6(6)
N2-N1-Cu1	116.5(4)	C4-C5-C6	122.2(6)
C7-N1-Cu1	125.6(5)	C1-C6-C5	119.2(6)
C7-N1-N2	117.7(6)	C1-C6-C7	123.9(5)
C8-N2-N1	119.3(6)	C7-C6-C5	116.8(5)
C8-N3-C9	122.4(7)	N1-C7-C6	125.1(6)
C11-N4-C10	110.3(5)	N2-C8-S1	120.6(6)
C11-N4-C12	109.5(6)	N3-C8-S1	122.2(6)
C11-N4-C13	110.9(6)	N3-C8-N2	117.2(7)
C12-N4-C10	107.7(5)	N3-C9-C14	109.8(7)
C12-N4-C13	108.5(5)	C4-C10-N4	115.4(5)
C13-N4C10	110.0(5)		

Table S3. Bond lengths [Å] and angles [°] for **4**.

**Cu1-Cl1**	**2.2653(11)**	**C1-C2**	**1.409(6)**
Cu1-S1	2.2562(12)	C1-C6	1.416(5)
Cu1-O1	1.908(3)	C2-C3	1.369(6)
Cu1-N1	1.971(3)	C3-C4	1.395(5)
S1-C8	1.712(4)	C4-C5	1.374(5)
O1-C1	1.311(4)	C4-C15	1.498(5)
N1-N2	1.385(4)	C5-C6	1.410(5)
N1-C7	1.290(5)	C6-C7	1.429(5)
N2-C8	1.339(5)	C9-C10	1.386(6)
N3-C8	1.317(5)	C9-C14	1.383(6)
N3-C9	1.432(5)	C10-C11	1.363(6)
N4-C15	1.519(5)	C11-C12	1.360(7)
N4-C16	1.496(5)	C12-C13	1.387(7)
N4-C17	1.491(5)	C13-C14	1.382(6)
N4-C18	1.486(5)		

S1-Cu1-Cl1	92.65(4)	C3-C2-C1	121.6(4)
O1-Cu1-Cl1	89.87(8)	C2-C3-C4	122.0(4)
O1-Cu1-S1	177.45(9)	C3-C4-C15	120.4(4)
O1-Cu1-N1	91.49(12)	C5-C4-C3	117.2(4)
N1-Cu1-Cl1	171.12(10)	C5-C4-C15	122.4(4)
N1-Cu1-S1	85.96(9)	C4-C5-C6	122.7(4)
C8-S1-Cu1	97.13(14)	C1-C6-C7	123.4(4)
C1-O1-Cu1	128.9(3)	C5-C6-C1	119.3(4)
N2-N1-Cu1	117.2(2)	C5-C6-C7	117.3(3)
C7-N1-Cu1	127.0(3)	N1-C7-C6	124.8(3)
C7-N1-N2	115.8(3)	N2-C8-S1	120.3(3)
C8-N2-N1	119.0(3)	N3-C8-S1	119.5(3)
C8-N3-C9	128.2(3)	N3-C8-N2	120.2(3)
C16-N4-C15	110.9(3)	C10-C9-N3	117.3(4)
C17-N4-C15	108.5(3)	C14-C9-N3	121.9(4)
C17-N4-C16	109.0(4)	C14-C9-C10	120.5(4)
C18-N4-C15	110.5(3)	C11-C10-C9	120.2(5)
C18-N4-C16	109.0(3)	C12-C11-C10	120.4(5)

C18-N4-C17	108.9(3)	C11-C12-C13	119.9(5)
O1-C1-C2	119.1(4)	C14-C13-C12	120.8(5)
O1-C1-C6	123.8(4)	C13-C14-C9	118.3(5)
C2-C1-C6	117.1(4)	C4-C15-N4	114.8(3)

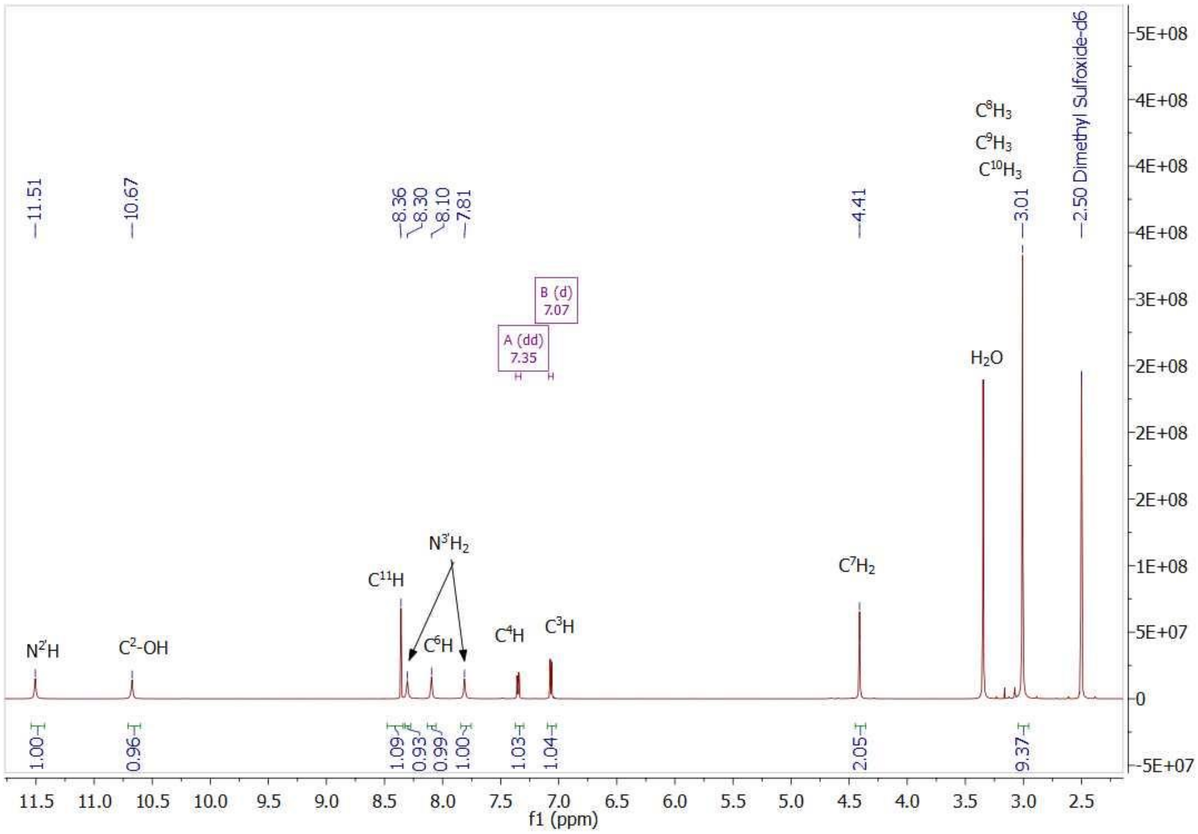

Figure 1. ¹H NMR spectrum of **[H₂L^H]Cl** in [D₆]DMSO.

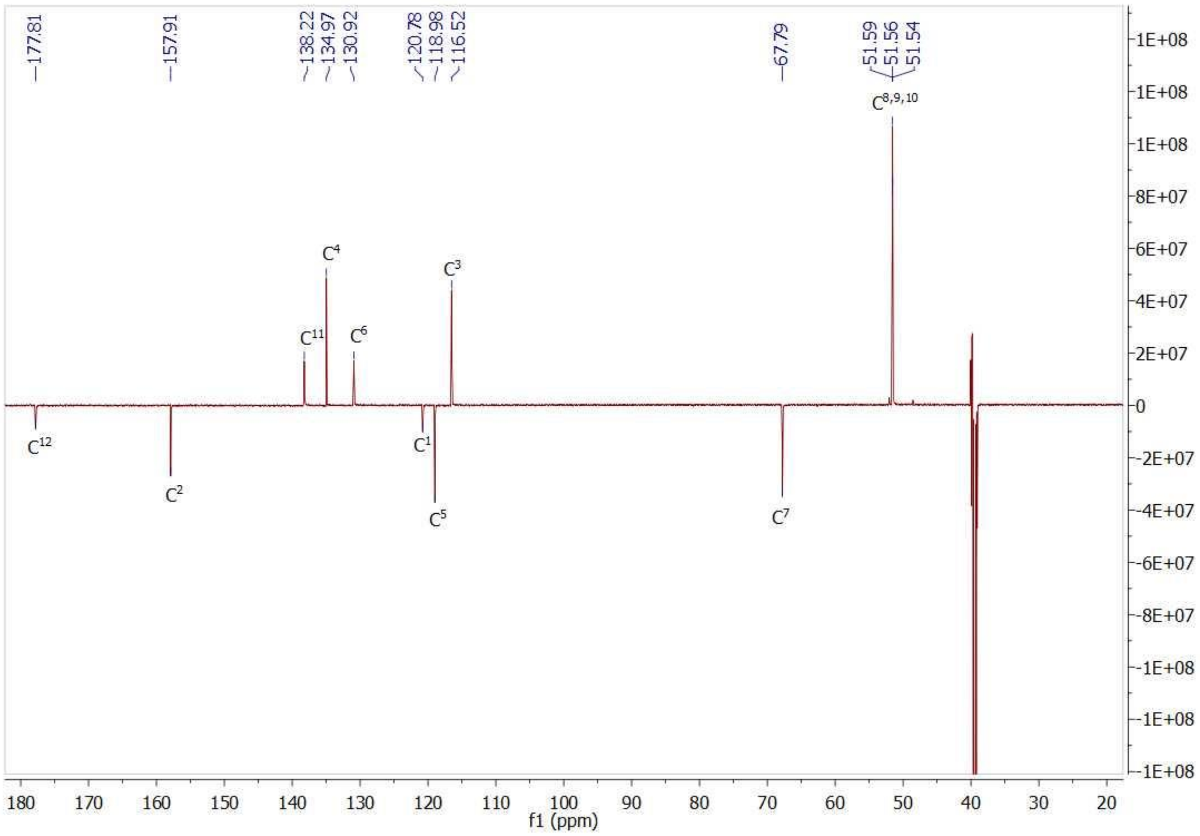

Figure 2. ^{13}C NMR spectrum of **[H$_2$L^H]Cl** in [D$_6$]DMSO.

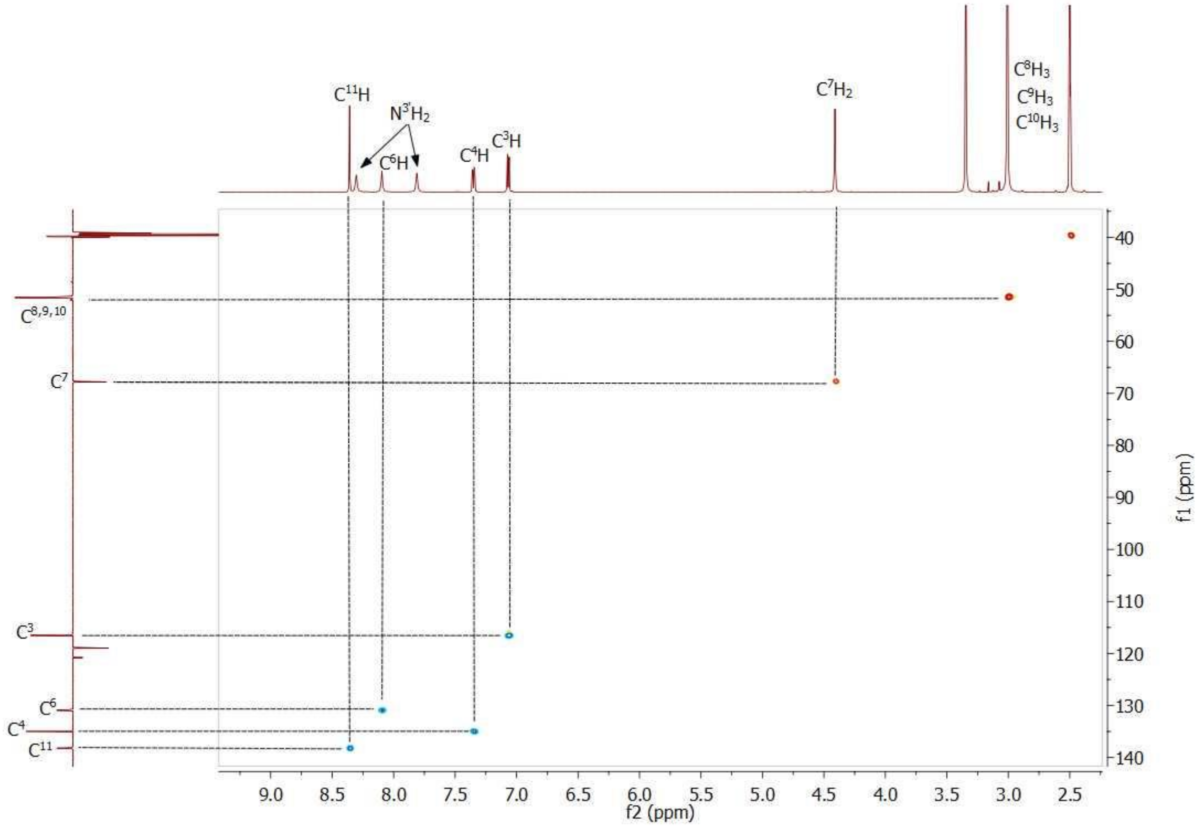

Figure 3. ^{1}H, ^{13}C HSQC NMR spectrum of **[H$_2$L^H]Cl** in [D$_6$]DMSO.

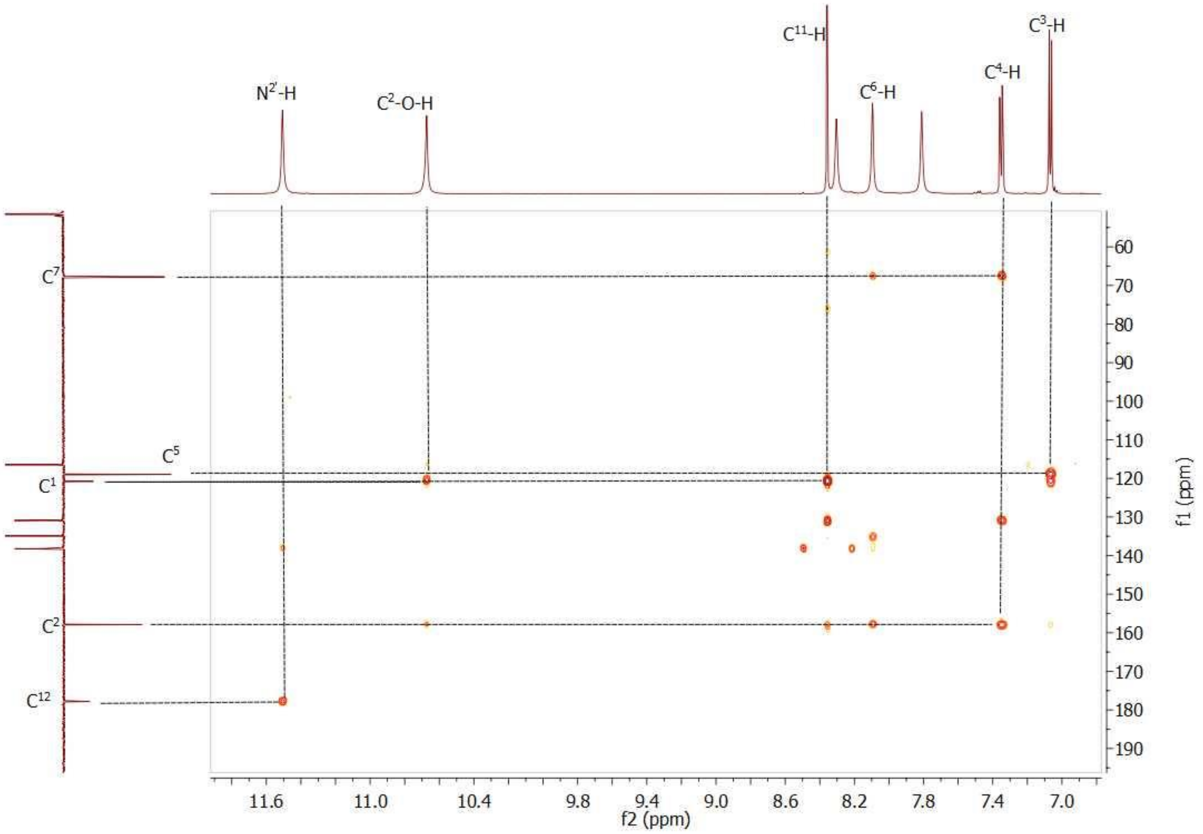

Figure 4. ^{1}H, ^{13}C HMBC NMR spectrum of **[H$_2$L^H]Cl** in [D$_6$]DMSO.

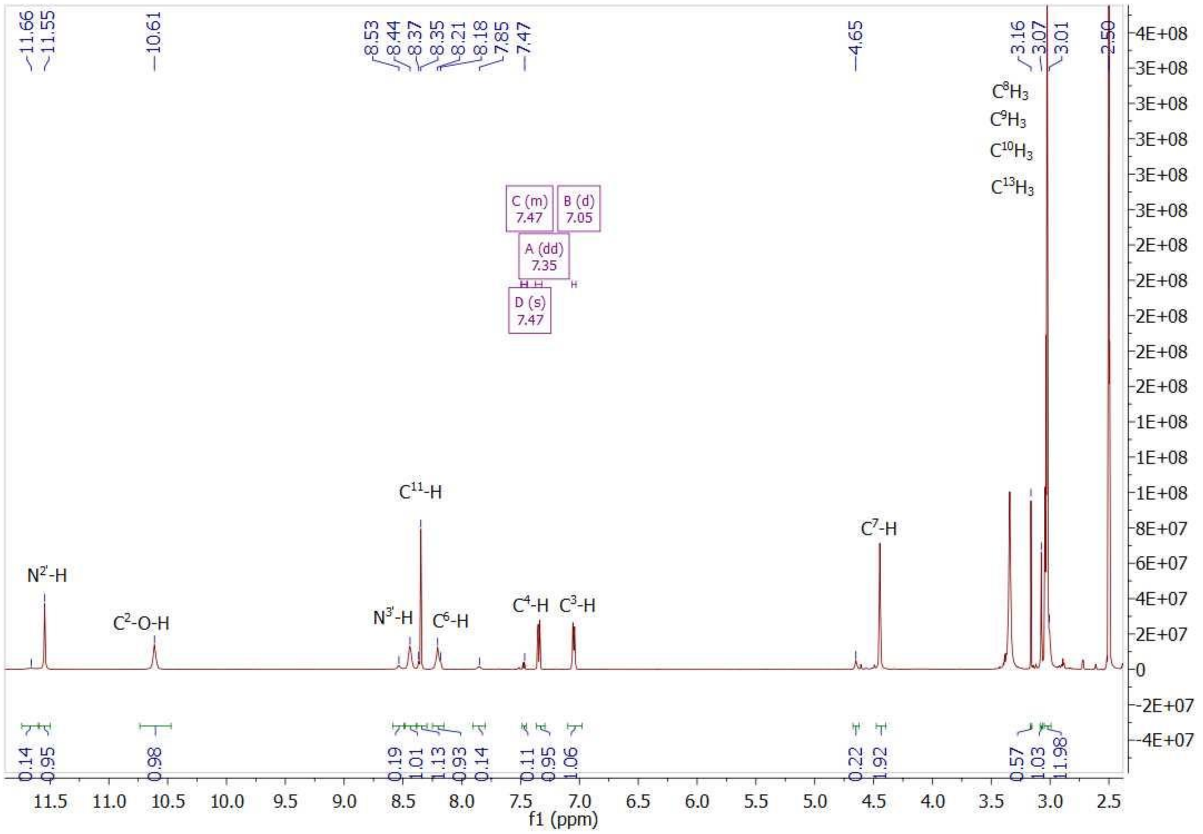

Figure 5. ^{1}H NMR spectrum of **[H$_2$L^{Me}]Cl** in [D$_6$]DMSO.

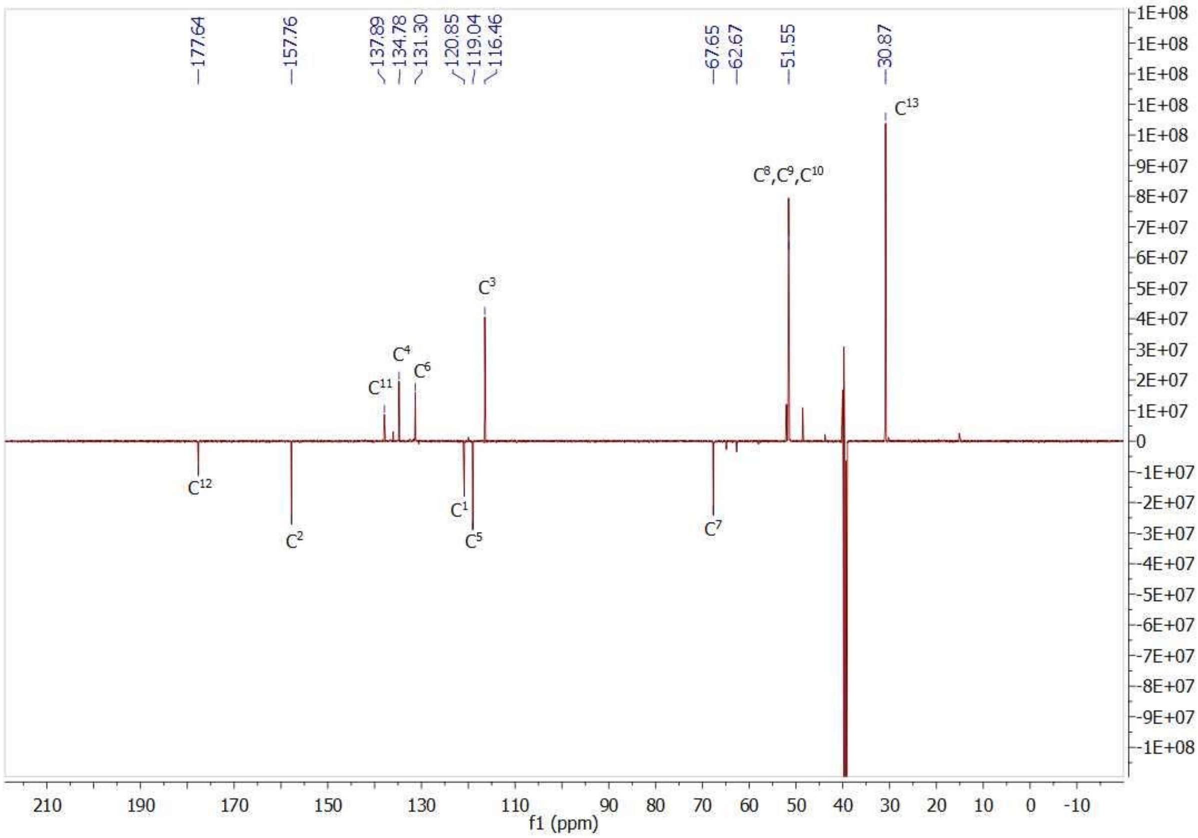

Figure 6. ^{13}C NMR spectrum of **[H$_2$L^{Me}]Cl** in [D$_6$]DMSO.

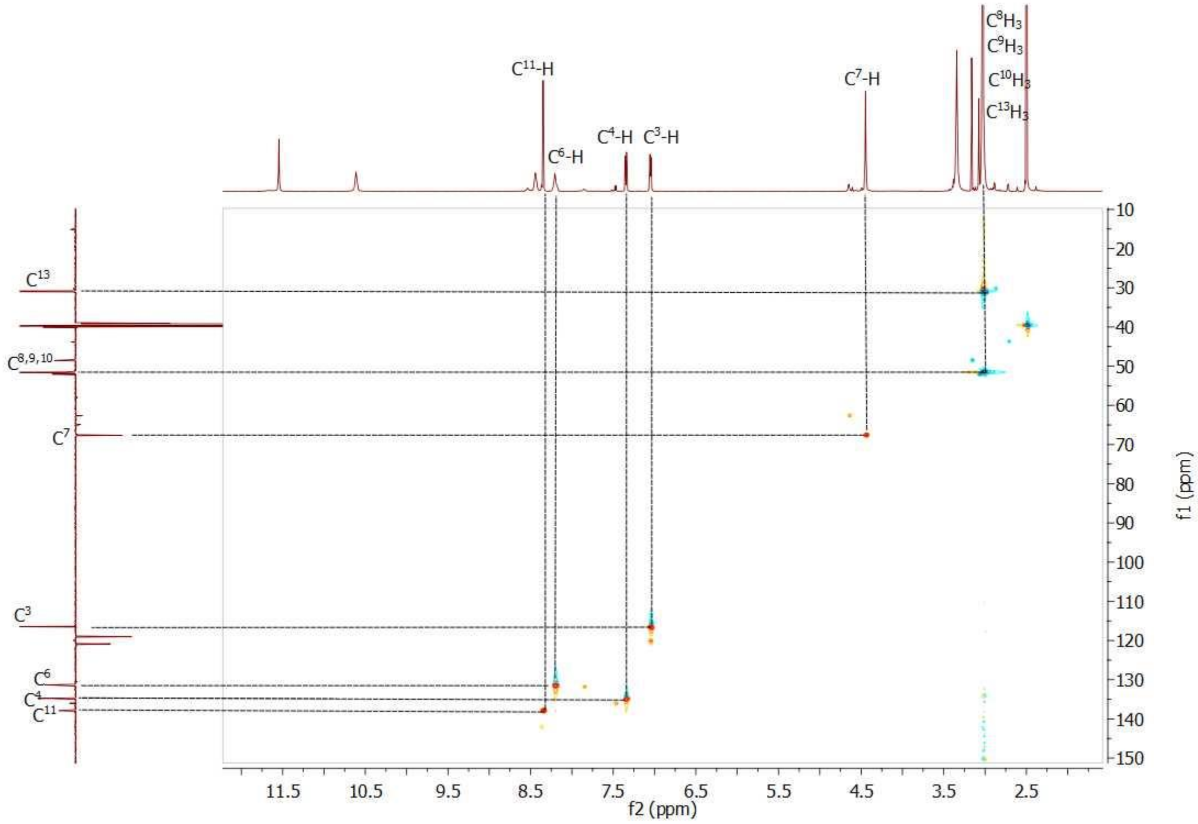

Figure 7. ^{1}H, ^{13}C HSQC NMR spectrum of **[H$_2$L^{Me}]Cl** in [D$_6$]DMSO.

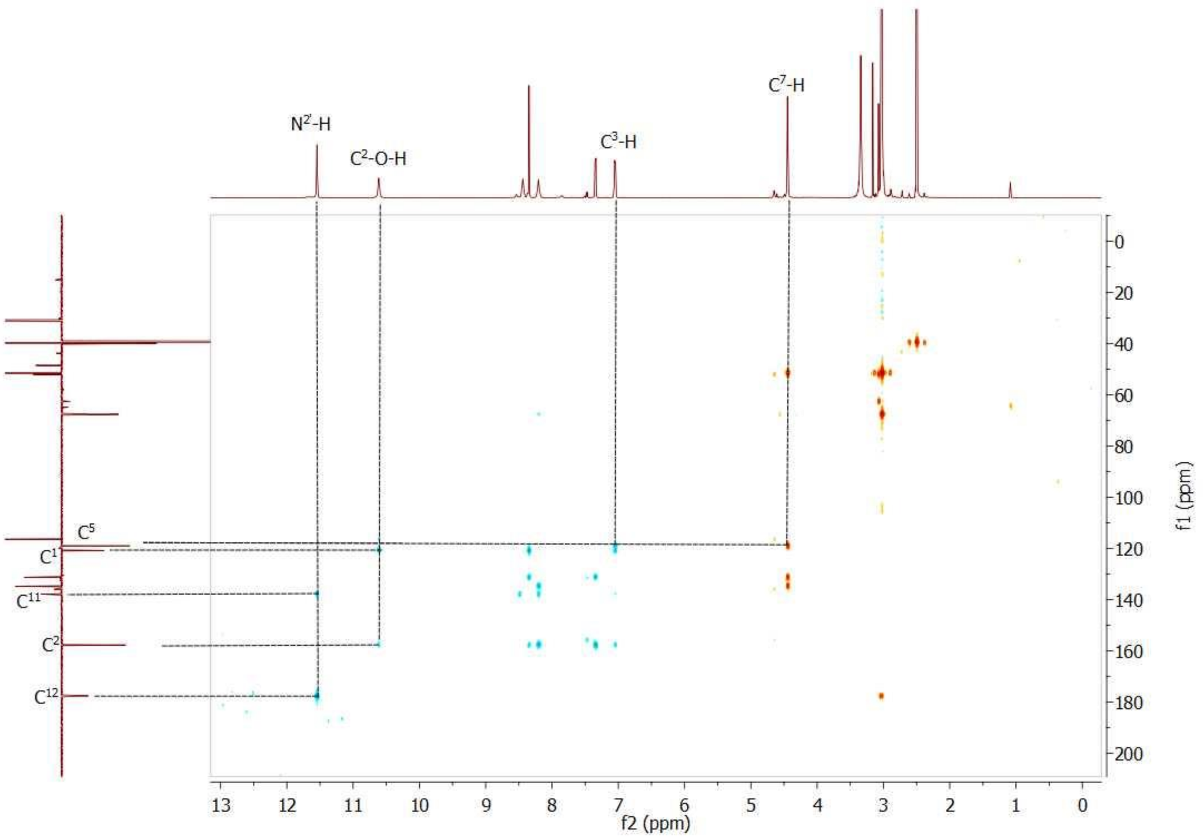

Figure 8. ^{1}H, ^{13}C HMBC NMR spectrum of **[H2L^{Me}]Cl** in [D6]DMSO.

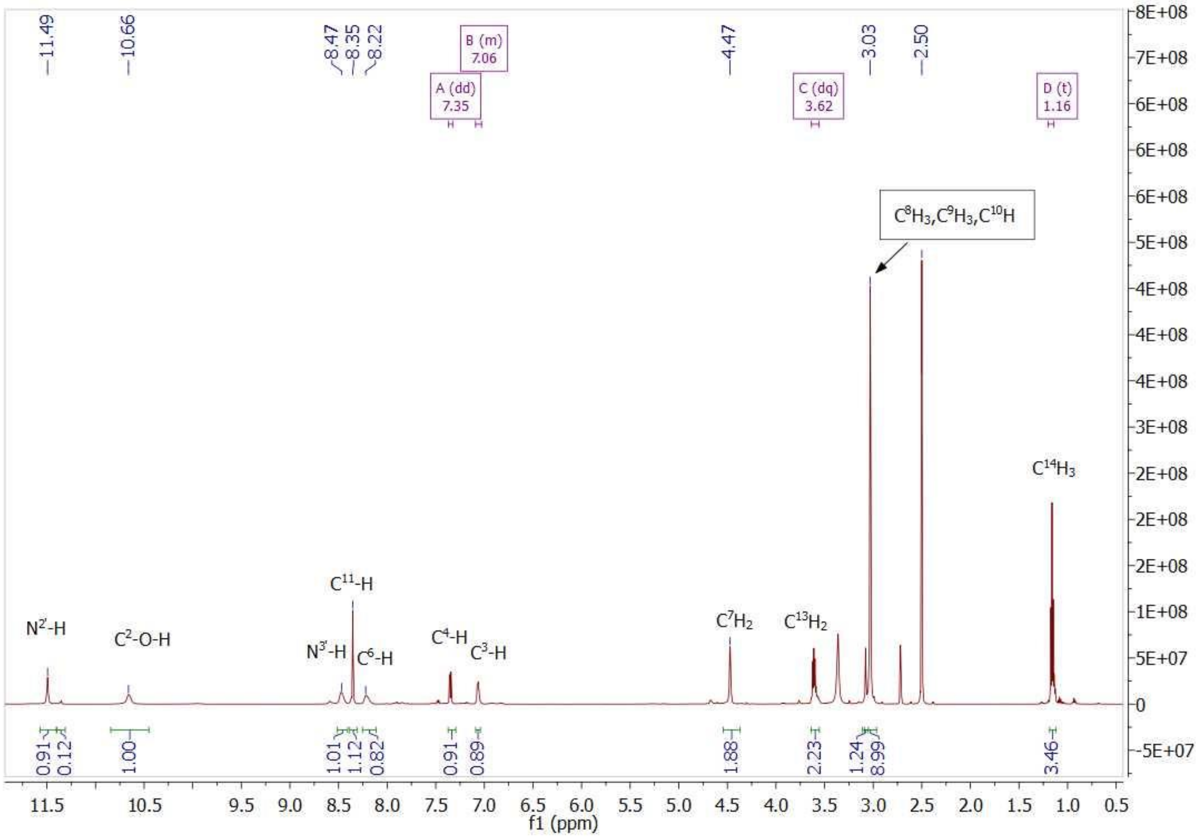

Figure 9. ^{1}H NMR spectrum of **[H2L^{Et}]Cl** in [D6]DMSO.

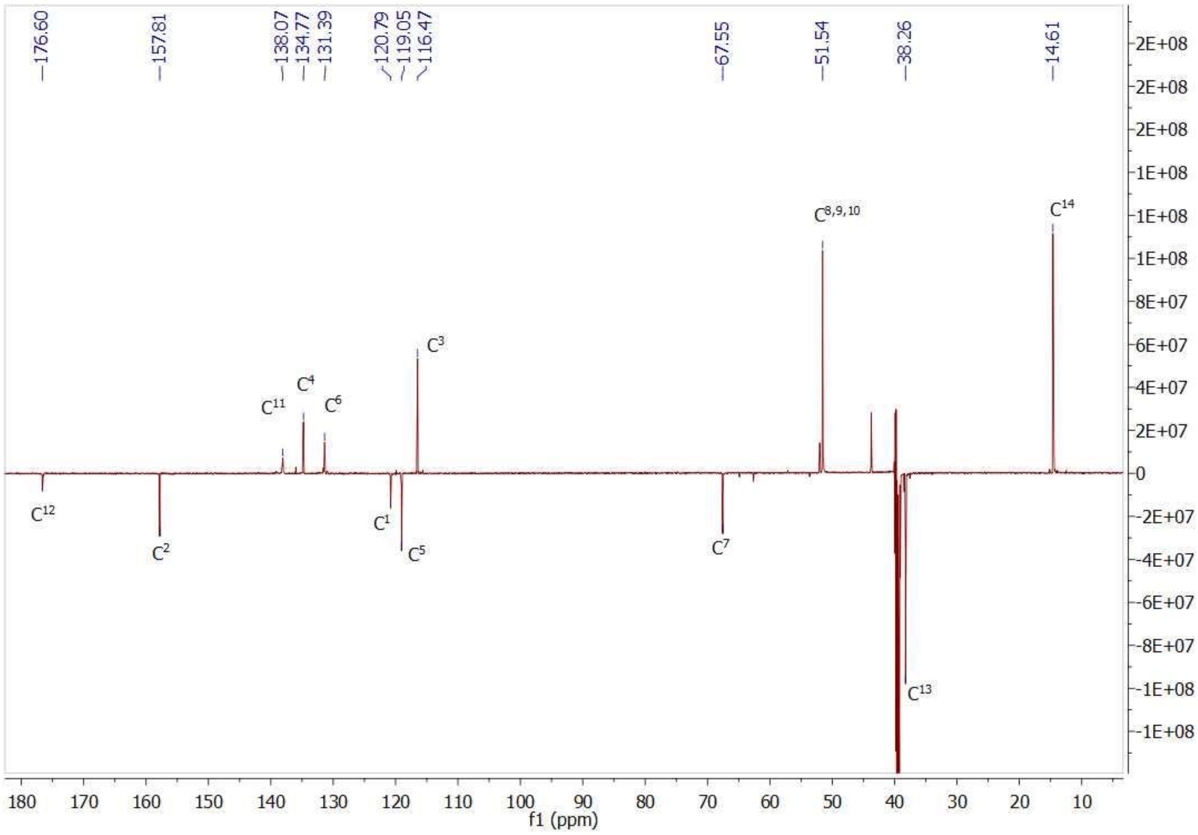

Figure 10. ^{13}C NMR spectrum of **[H$_2$L^{Et}]Cl** in [D$_6$]DMSO.

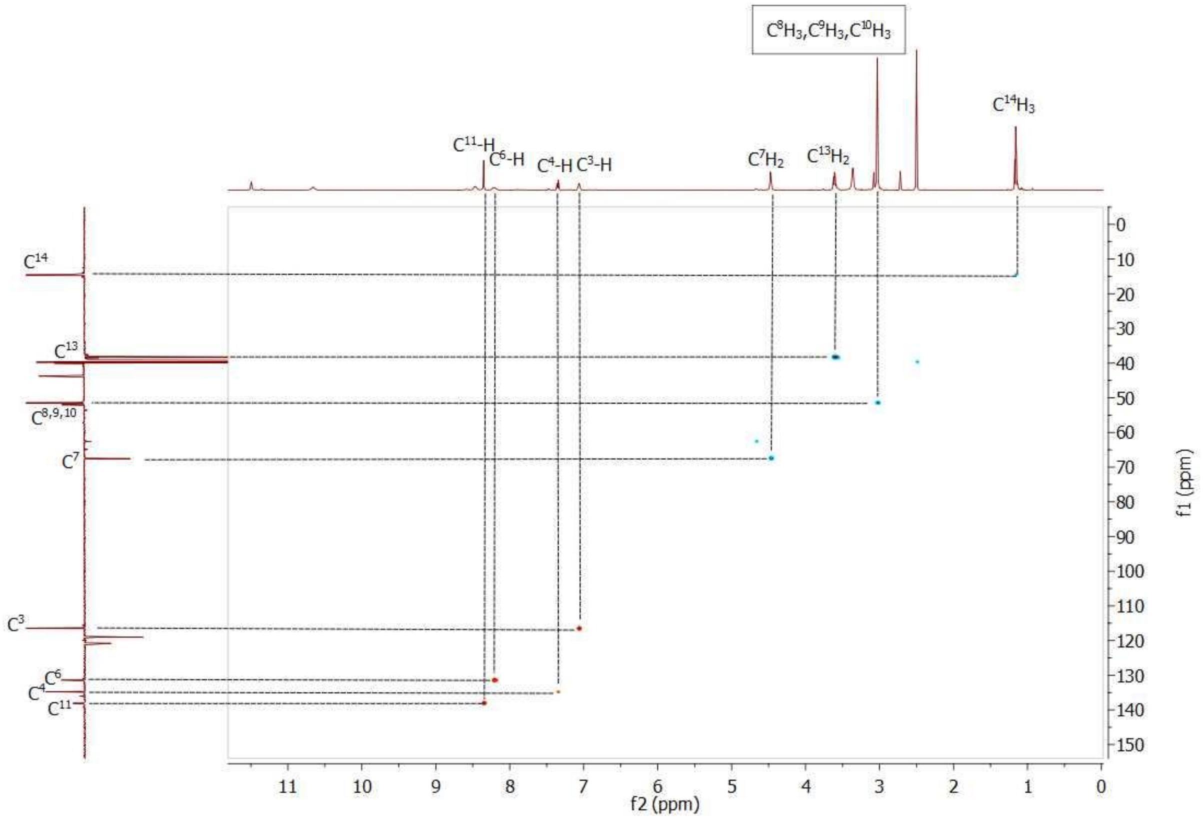

Figure 11. ^{1}H, ^{13}C HSQC NMR spectrum of **[H$_2$L^{Et}]Cl** in [D$_6$]DMSO.

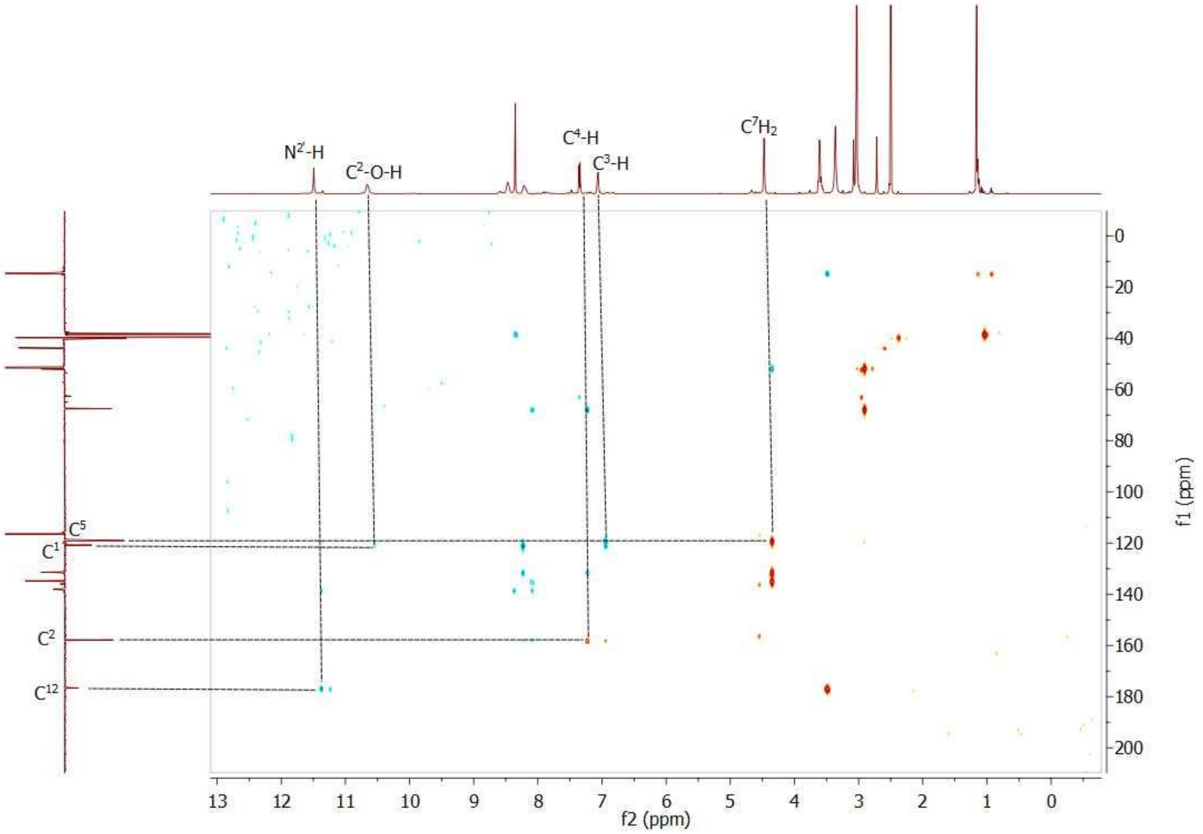

Figure 12. ^{1}H, ^{13}C HMBC NMR spectrum of **[H$_2$L^{Et}]Cl** in [D$_6$]DMSO.

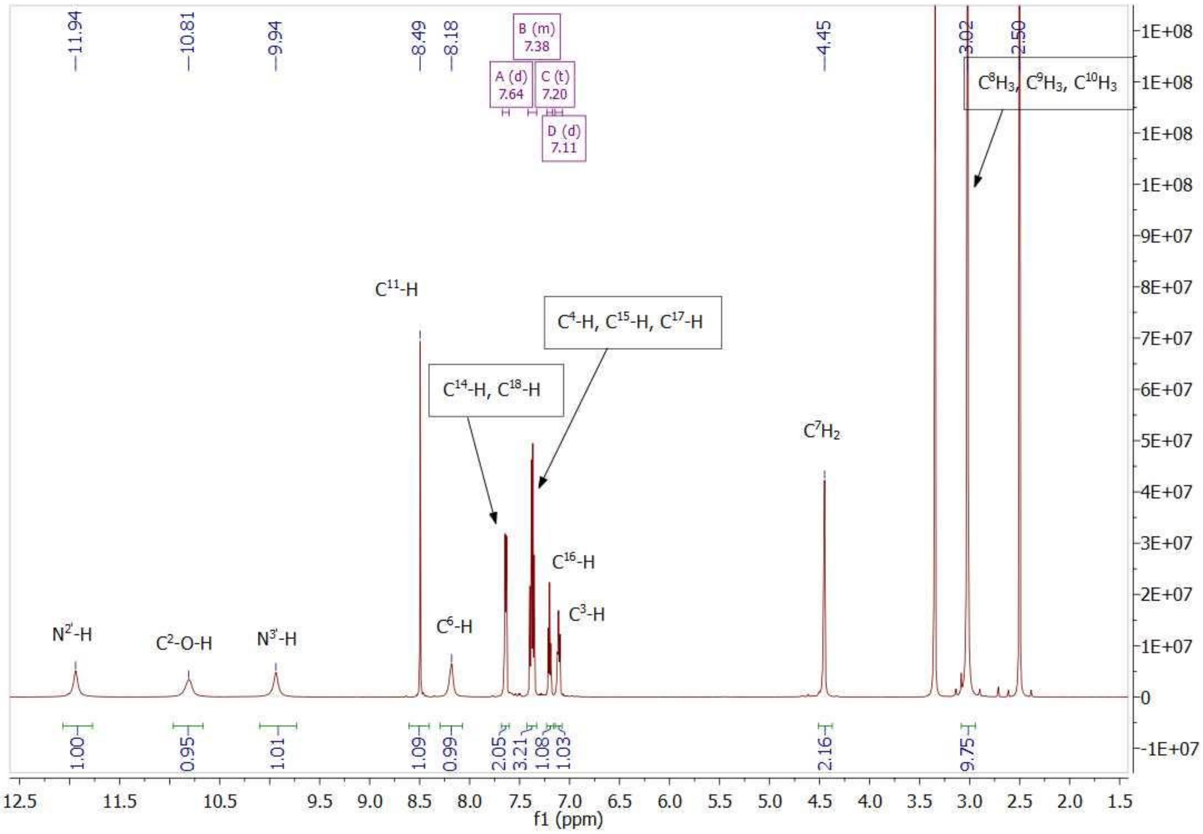

Figure 13. ^{1}H NMR spectrum of **[H$_2$L^{Ph}]Cl** in [D$_6$]DMSO.

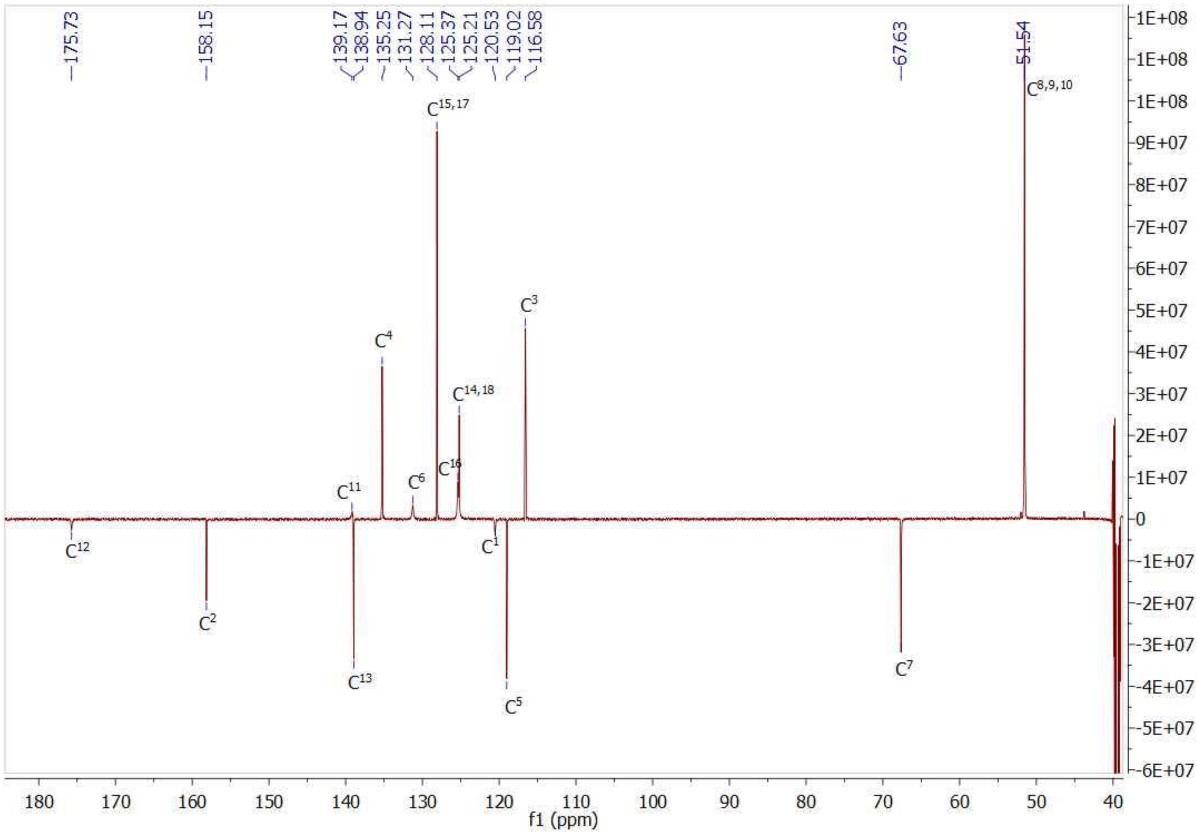

Figure 14. ^{13}C NMR spectrum of **[H$_2$L^{Ph}]Cl** in [D$_6$]DMSO.

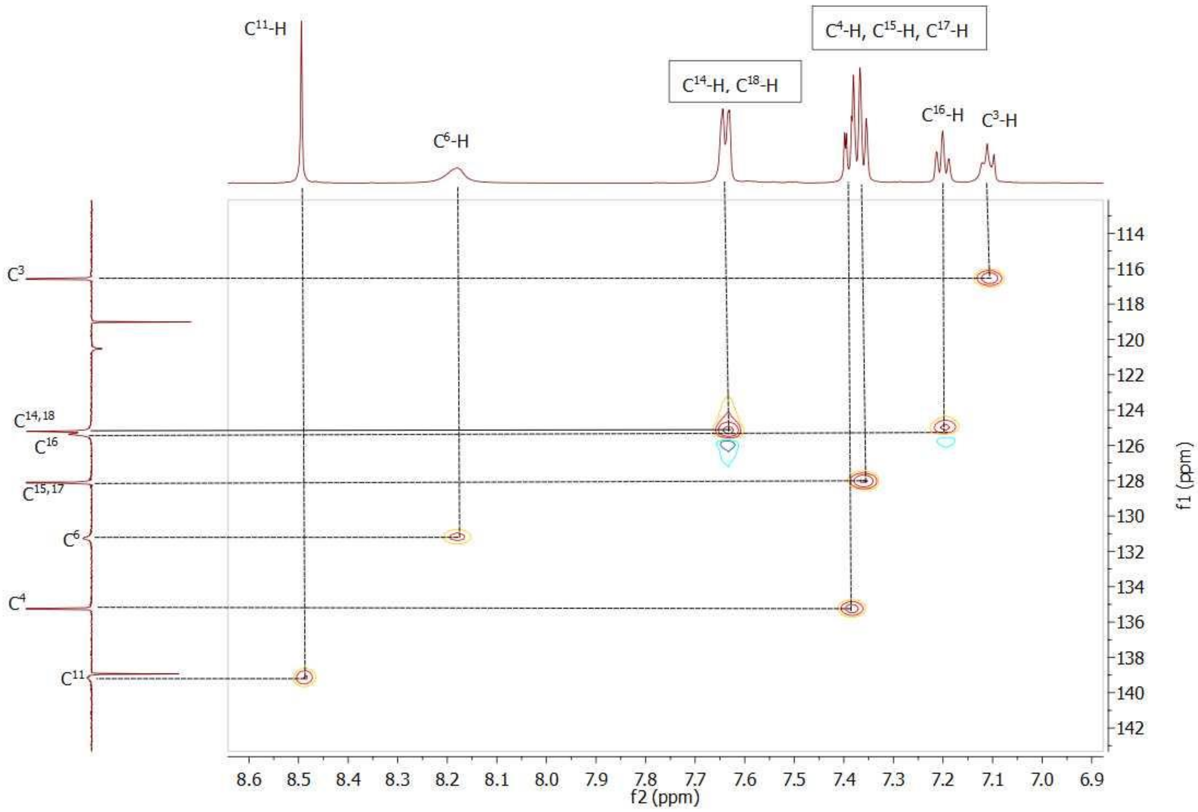

Figure 15. ^{1}H, ^{13}C HSQC NMR spectrum of **[H$_2$L^{Et}]Cl** in [D$_6$]DMSO.

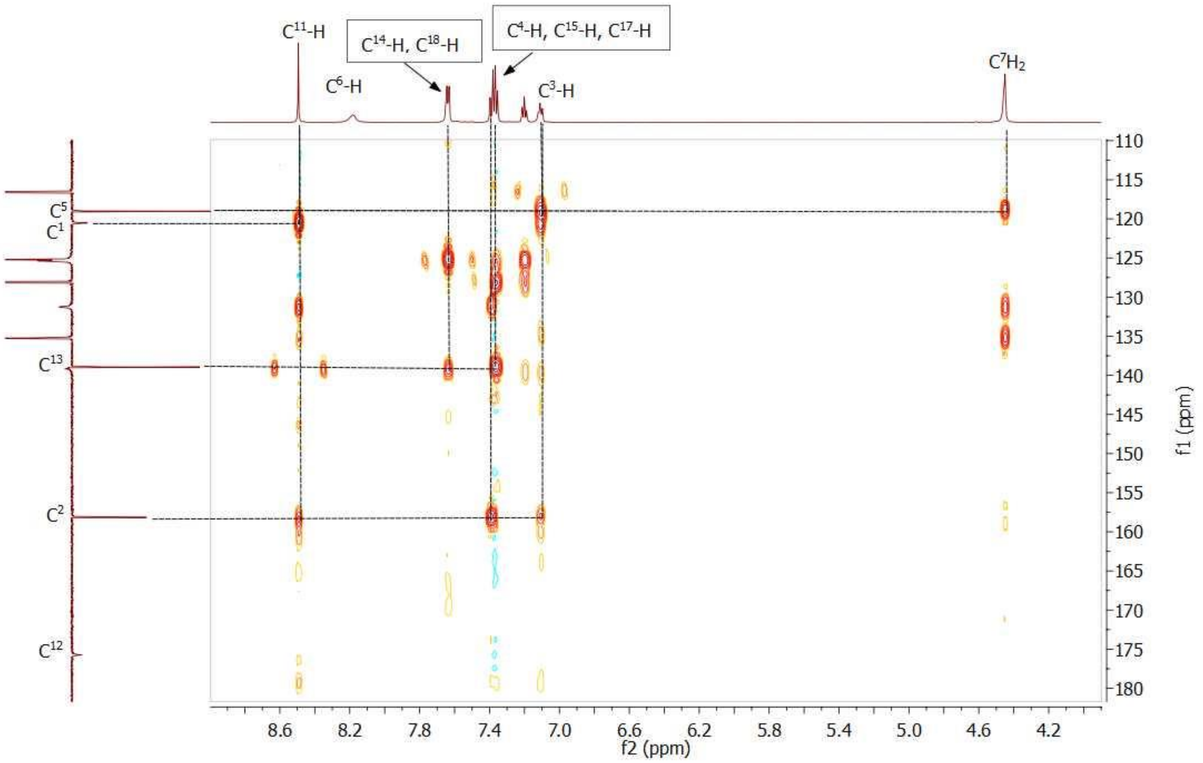

Figure 16. The fragment of ^{1}H, ^{13}C HMBC NMR spectrum of **[H_2L^{Ph}]Cl** in [D_6]DMSO.

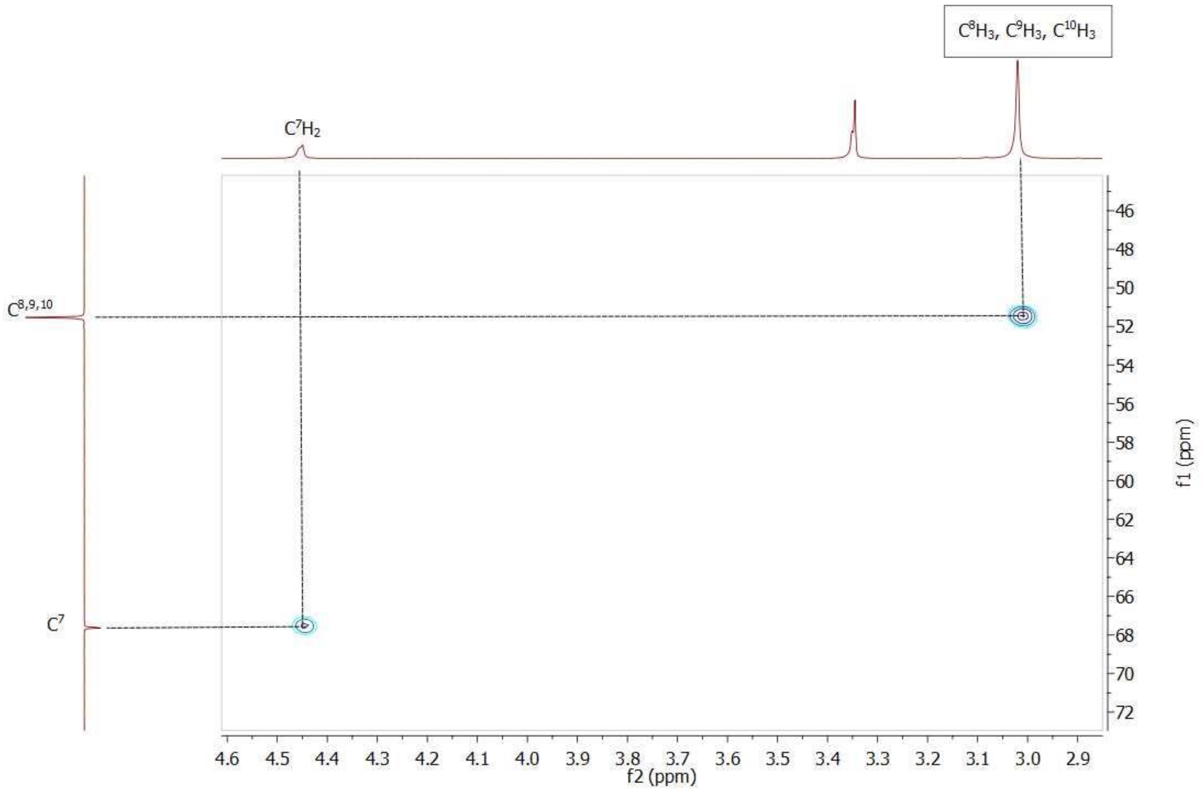

Figure 17. The fragment of ^{1}H, ^{13}C HMBC NMR spectrum of **[H_2L^{Ph}]Cl** in [D_6]DMSO.

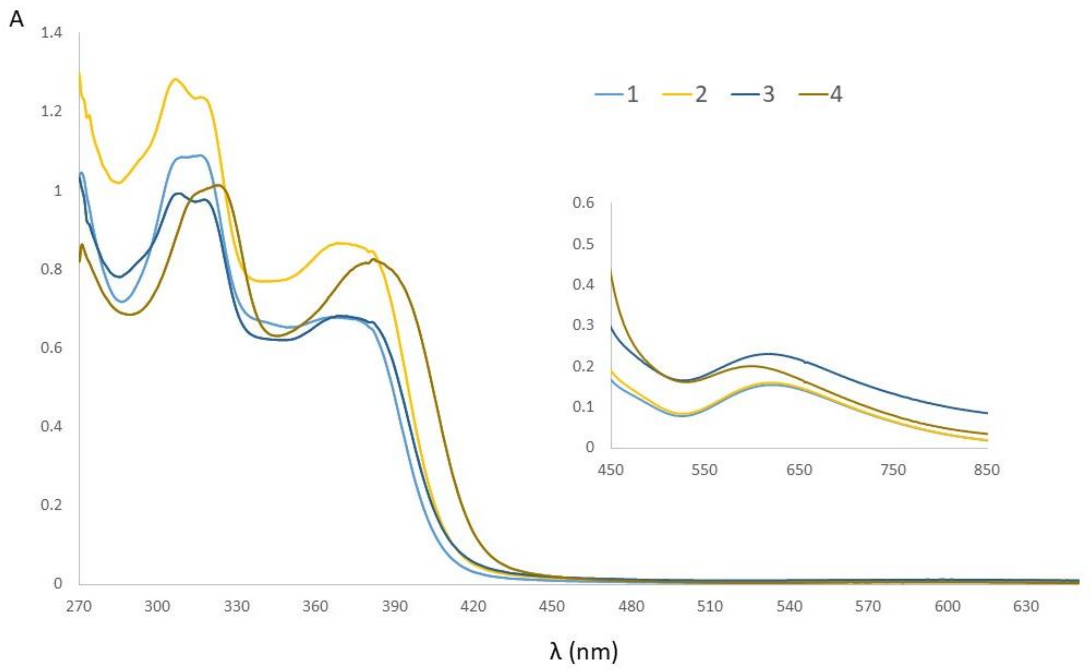

Figure 18. UV/Vis absorption spectra of copper(II) complexes **1** (c_1 = 59 μM), **2** (c_2 = 51 μM), **3** (c_3 = 56 μM) and **4** (c_4 = 50 μM) in aqueous solutions (path length 1 cm) .

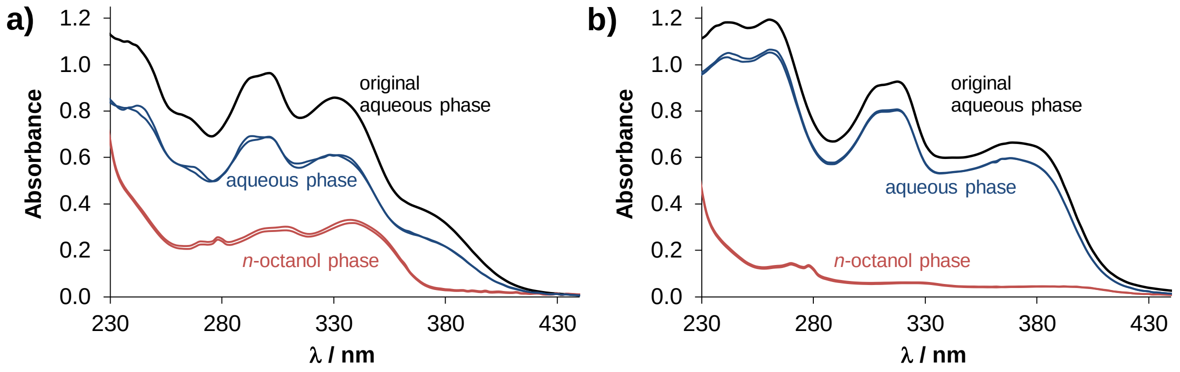

Figure 19. (a) UV/Vis absorption spectra recorded for proligand **[H_2L^{Me}]Cl** and (b) its copper(II) complex **2** in aqueous solution before (black line) and after (blue lines) partitioning and in the *n*-octanol phase (red lines) after the partitioning (pH = 7.4 (20 mM PBS); I = 0.1 M (KCl); t = 25 °C).

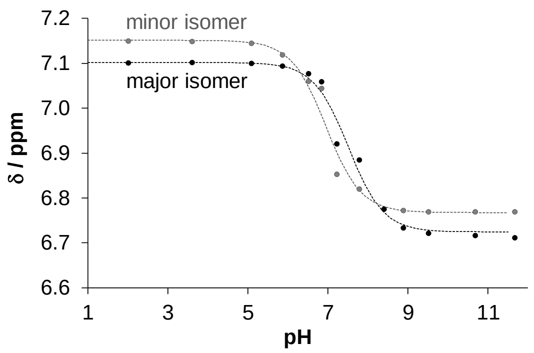

Figure 20. Chemical shifts of the CH(4) proton of the major (●) and minor (●) isomers of **[H_2L^H]Cl** plotted against the pH values in addition to the fitted curves (dashed lines) (c_L = 200 μM; I = 0.1 M (KCl); t = 25 °C; 10% (v/v) D_2O/H_2O).

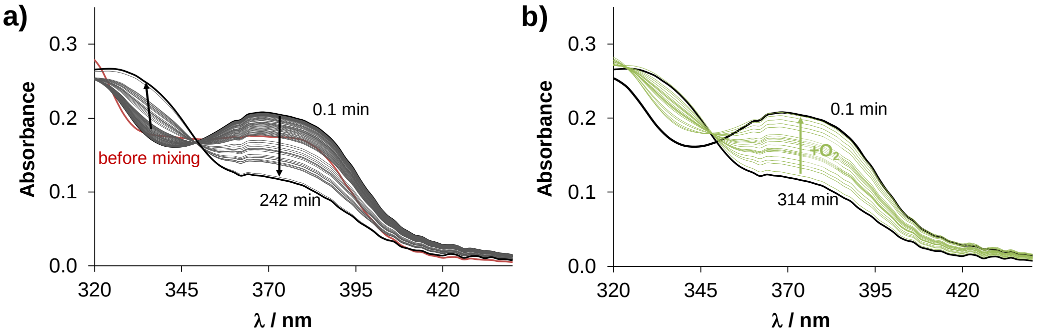

Figure 21. (a) Time-dependent UV/Vis absorption spectra of complex **1** in the presence of 50 equiv GSH before (red line) and after mixing their solutions (black lines) in a tandem cuvette. (b) Absorbance changes after bubbling oxygen into the reaction mixture (green lines); (pH = 7.4 (50 mM HEPES); $c_{complex}$ = 25 µM; c_{GSH} = 1.25 mM; I = 0.1 M (KCl); t = 25 °C).

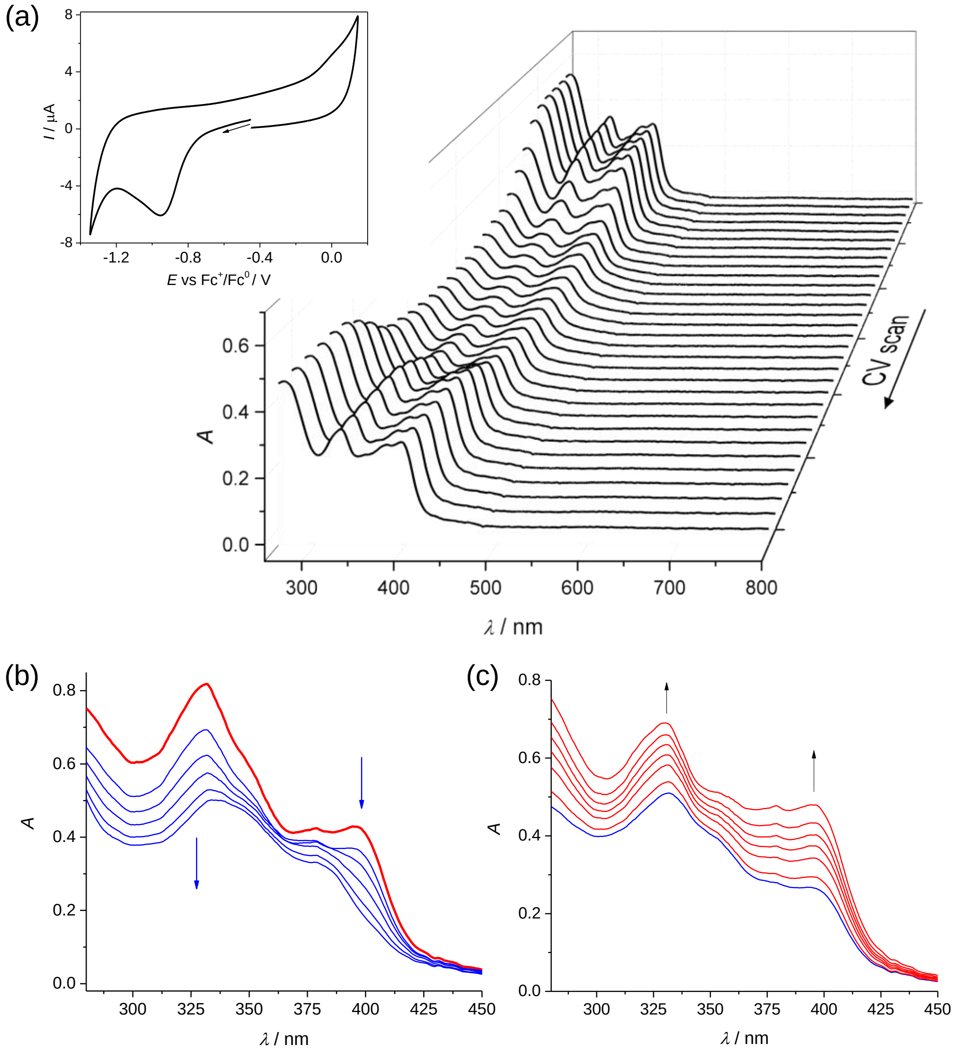

Figure 22. (a) UV/Vis spectra measured during cyclic voltammetry of **2** in MeCN/0.1 M nBu$_4$NPF$_6$ (Inset: the corresponding in situ cyclic voltammogram) and (b) during prolonged cathodic reduction of **3** in MeCN/0.1 M nBu$_4$NPF$_6$ at the first reduction peak and (c) their recovery observed upon reoxidation during reverse scan (scan rate 10 mV s^{-1}).

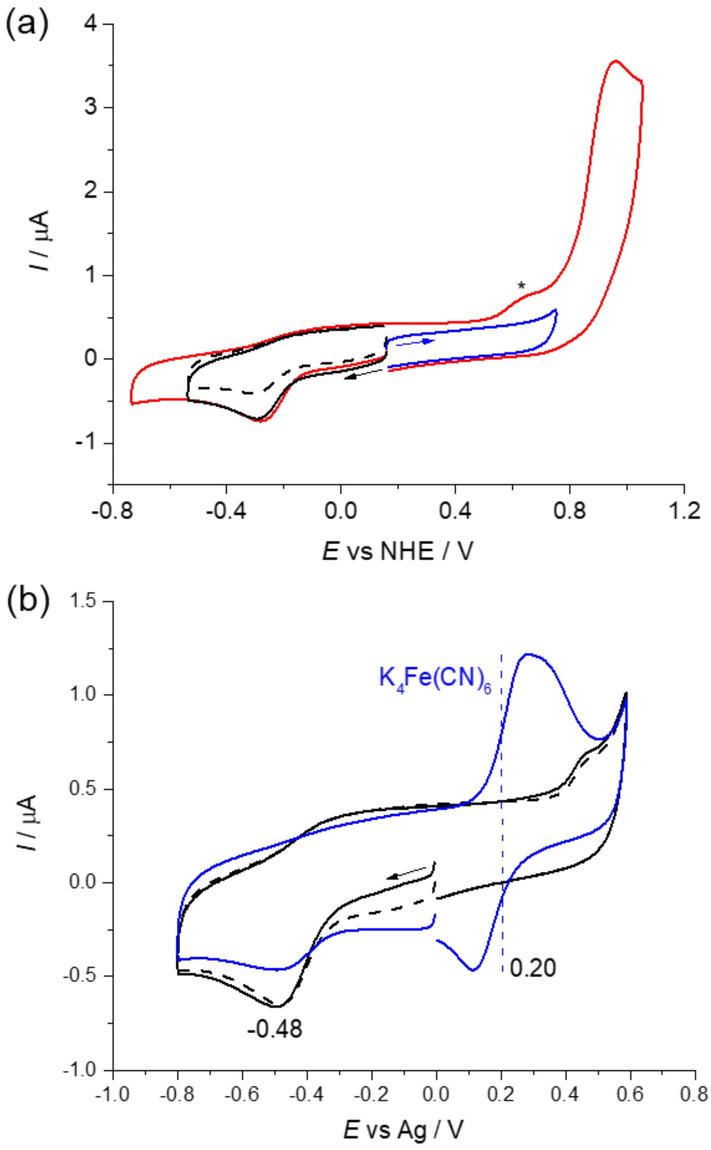

Figure 23. Cyclic voltammograms of (a) **1** and (b) **3** in phosphate buffer in the presence of 0.1 M KCl (pH 7) using a glassy carbon disc electrode at scan rate of 100 mV s^{-1}.

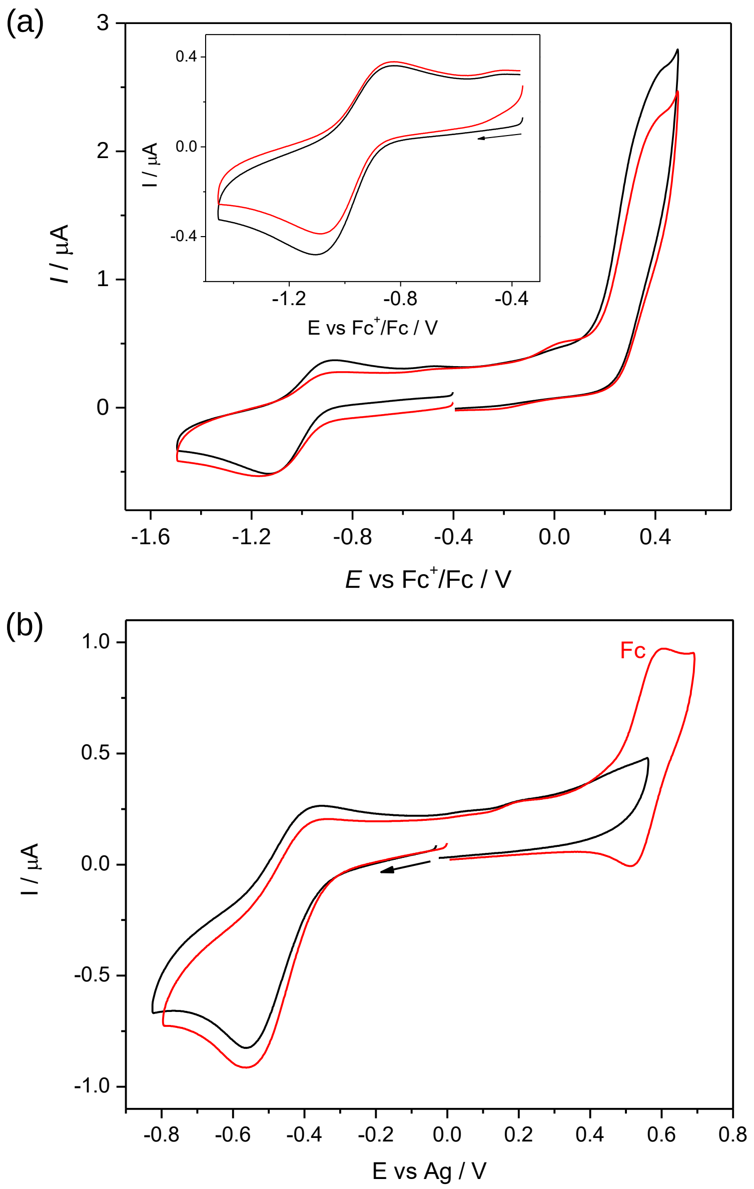

Figure 24. (a) Cyclic voltammograms of **2** in DMSO/*n*Bu_4NPF_6 using a glassy carbon disc working electrode. Inset: the cyclic voltammograms in the cathodic part (two consecutive scans, scan rate of 100 mV s^{-1}). (b) Cyclic voltammograms of **2** in DMSO/*n*Bu_4NPF_6 using a Pt-disc working electrode in the absence (black line) and in the presence (red line) of ferrocene.

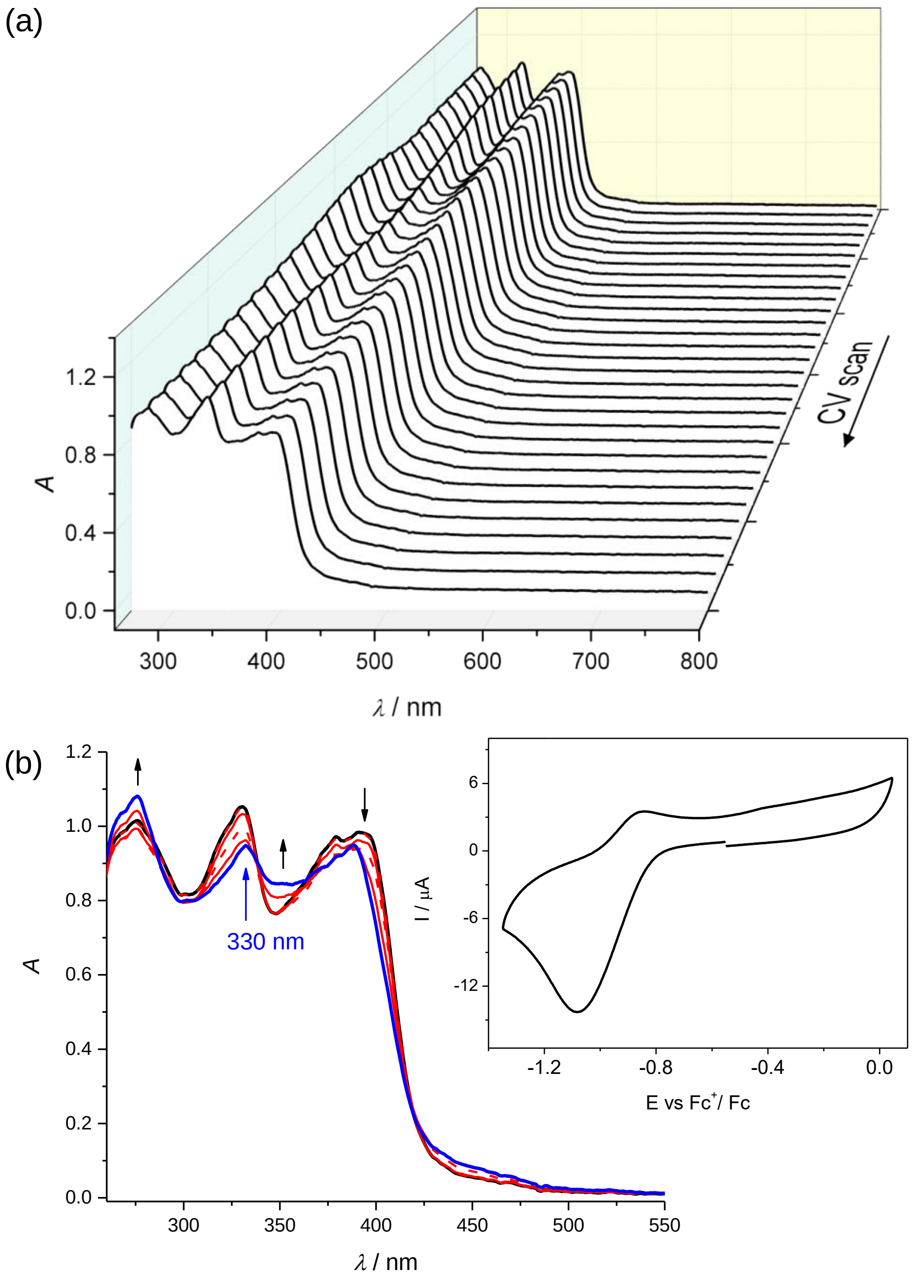

Figure 25. (a) UV/Vis spectra measured during cyclic voltammetry of **2** in DMSO/0.1 M *n*Bu_4NPF_6. (b) Selected UV/Vis spectra measured during the forward scan. Inset: the corresponding in situ cyclic voltammogram (scan rate 10 mV s^{-1}).

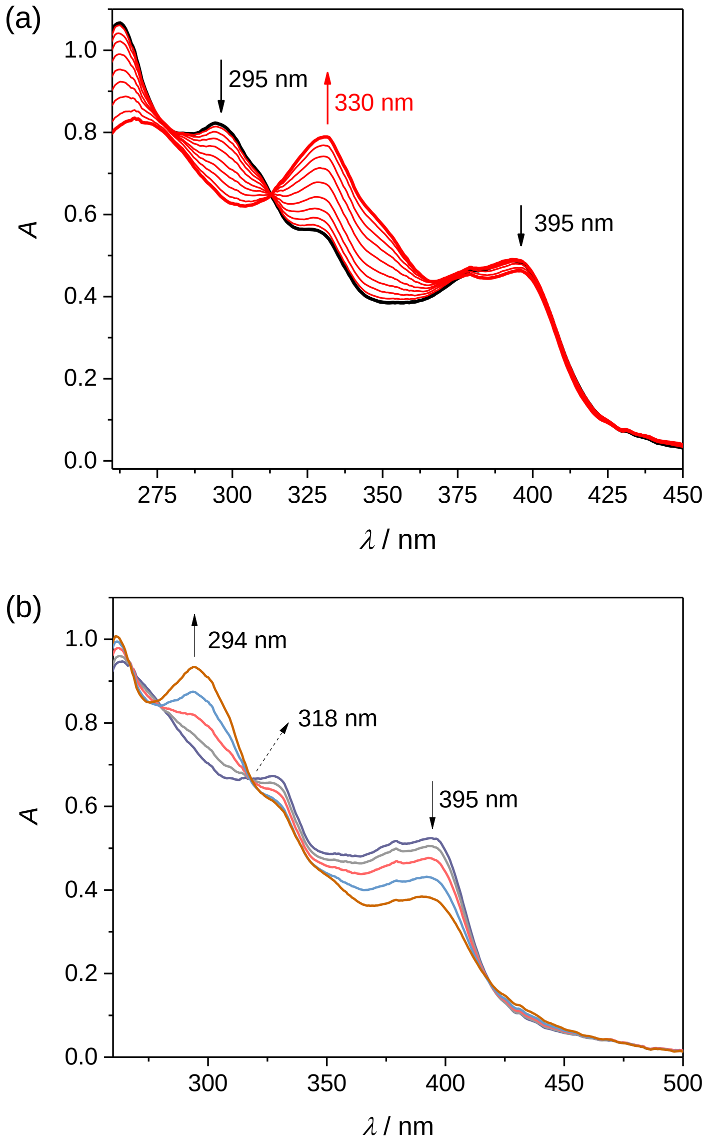

Figure 26. UV/Vis spectra measured upon (a) cathodic reduction and (b) anodic oxidation of **3** in MeCN/0.1 M *n*Bu_4NPF_6 (forward scans, scan rate 10 mV s^{-1}).

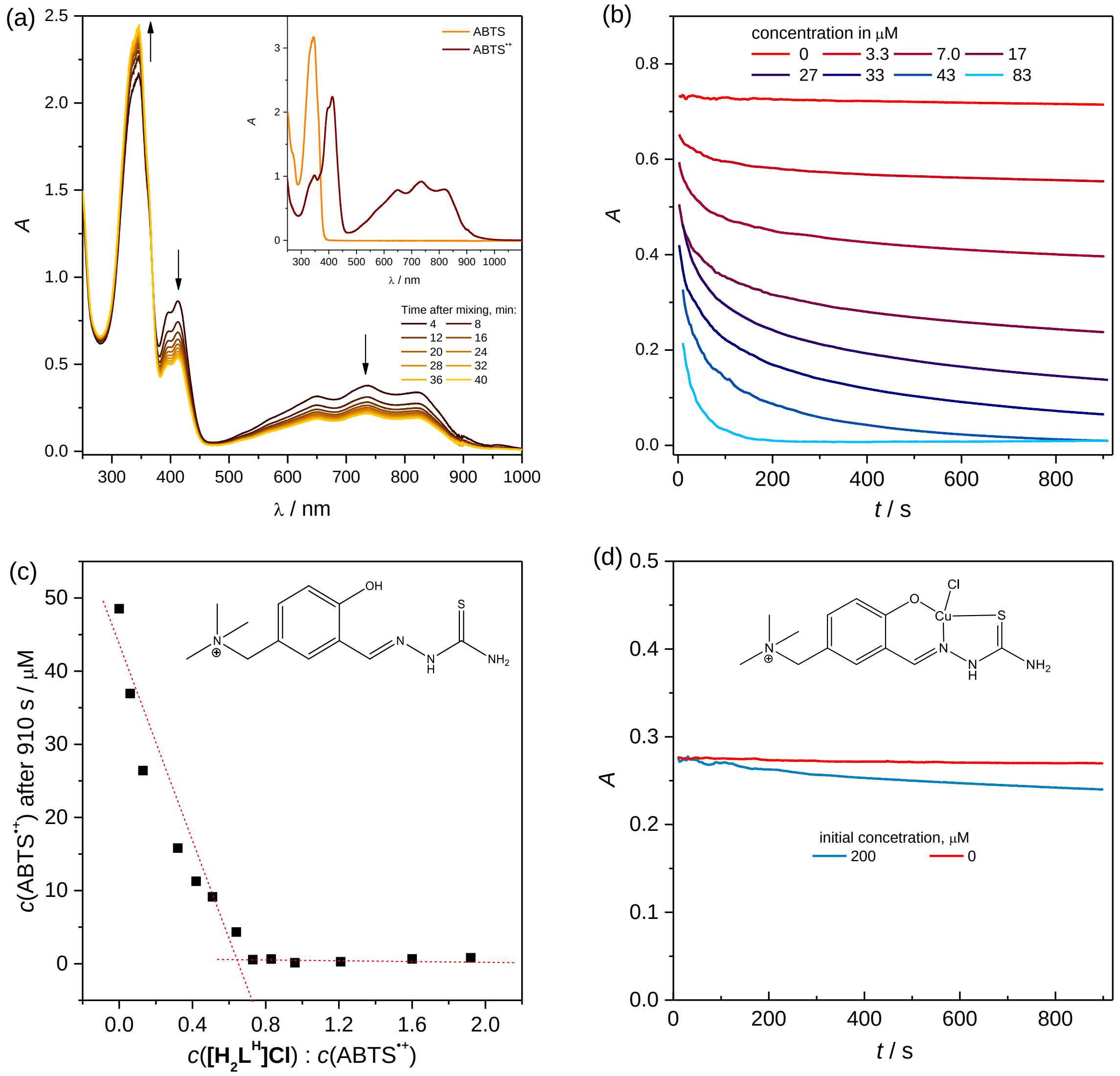

Figure 27. (a) Set of electronic absorption spectra of ABTS$^{\bullet+}$measured after mixing in **[H$_2$L^H]Cl** system (c_{in}(ABTS$^{\cdot+}$) = 48.5 μM; $c_{in,[H2L^H]Cl}$ = 10 μM, DMSO content 20% (v/v)); inset in (a) represents the electronic absorption spectra of ABTS$^{\bullet+}$ radical cation and its parent molecule ABTS in mixed solvent water: DMSO (20% (v/v)); (b) Changes in ABTS$^{\bullet+}$ absorption at 735 nm monitored after mixing **[H$_2$L^H]Cl** DMSO solutions with ABTS$^{\bullet+}$ (c_{in}(ABTS$^{\bullet+}$) = 48.5 μM; The initial concentrations of **[H$_2$L^H]Cl** are marked in Figure; DMSO content 20% (v/v)); (c) Concentration of ABTS$^{\bullet+}$ measured 910 s after mixing with **[H$_2$L^H]Cl** quoted upon increasing initial molar ratios **[H$_2$L^H]Cl** /ABTS$^{\bullet+}$; (d) Changes in ABTS$^{\bullet+}$ absorption at 735 nm monitored after mixing DMSO solutions of **1** with ABTS$^{\bullet+}$ (c_{in}(ABTS$^{\bullet+}$) = 18 μM; The initial concentrations of **1** are indicated in the Figure; DMSO content 20% (v/v)).
